# The association between lifelines diet score and metabolic associated fatty liver disease: a case–control study

**DOI:** 10.3389/fnut.2025.1569814

**Published:** 2025-05-15

**Authors:** Thanyaporn Direksunthorn, Amr Ali Mohamed Abdelgawwad El-Sehrawy, Ahmed Hjazi, Safia Obaidur Rab, Marwah Suliman Maashi

**Affiliations:** ^1^School of Medicine, Walailak University, Tha Sala District, Surat Skin Clinic, Surat Thani, Thailand; ^2^Department of internal Medicine, Diabetes, Endocrinology and Metabolism, Mansoura University, Mansoura, Egypt; ^3^Department of Medical Laboratory, College of Applied Medical Sciences, Prince Sattam bin Abdulaziz University, Al-Kharj, Saudi Arabia; ^4^Central Labs, King Khalid University, AlQura’a, Abha, Saudi Arabia; ^5^Department of Clinical Laboratory Sciences, College of Applied Medical Sciences, King Khalid University, Abha, Saudi Arabia; ^6^Medical Laboratory Sciences Department, Faculty of Applied Medical Sciences, King Abdulaziz University, Jeddah, Saudi Arabia; ^7^Regenerative Medicine Unit at King Fahd Medical Research Center, Jeddah, Saudi Arabia

**Keywords:** lifelines diet score, LLDS, metabolic associated fatty liver disease, MAFLD, diet

## Abstract

**Introduction:**

Adherence to a healthy dietary pattern is a fundamental recommendation for the prevention of Metabolic Associated Fatty Liver Disease (MAFLD); however, conclusive evidence regarding the optimal dietary pattern remains elusive.

**Objectives:**

The Lifelines Diet Score (LLDS) is a novel, evidence-based scoring system designed to evaluate diet quality. However, despite the extensive research on dietary patterns and liver health, the specific relationship between the LLDS and MAFLD remains underexplored. This study aims to investigate the association between LLDS and MAFLD, providing insights into how dietary adherence, as measured by LLDS, may influence the risk and prevalence of MAFLD.

**Methods:**

This case–control study enrolled 215 individuals who had recently been diagnosed with MAFLD and 430 healthy controls at King Khalid University Hospital. All participants were aged between 20 and 60 years, with data collection occurring from February 2023 to January 2025. The dietary intake of the participants was assessed through the utilization of a validated semi-quantitative food frequency questionnaire, which comprised a total of 168 distinct food items. Logistic regression was used to estimate the association between LLDS and MAFLD.

**Results:**

Out of 645 participants, 215 newly diagnosed MAFLD patients and 430 healthy controls were analyzed. After stratifying participants based on LLDS tertiles, those in the highest LLDS group had a 78% lower odds of MAFLD than those in the lowest tertile (odds ratio (OR): 0.22; 95% Confidence interval (CI): 0.12–0.36, *p* for trend <0.001). The association remained robust even after adjustment for major confounders. These findings highlight a novel and robust association between LLDS and MAFLD, providing evidence for dietary pattern assessment in liver health research.

**Conclusion:**

Our study strengthens the evidence that adherence to a healthy dietary pattern (as measured by LLDS) is associated with a lower MAFLD risk, even after accounting for major confounders. However, further research integrating genetic and molecular data is needed to refine personalized dietary recommendations for MAFLD prevention.

## 1 Introduction

Metabolic Associated Fatty Liver Disease (MAFLD) is a spectrum of hepatic conditions in which hepatocytes become overloaded with excessive adipose tissue, not due to ethanol consumption. Adiposity, type 2 diabetes mellitus and dyslipidemia are closely associated with MAFLD (1). The recent scientific literature redefines NAFLD as MAFLD, based on the metabolic basis of the disease and better defines its pathophysiology. Adiposity is a condition that is rapidly becoming common worldwide as rates of adiposity increase (2). Understanding MAFLD is crucial because it can progress to more severe liver diseases, including steatohepatitis, fibrosis, cirrhosis, and hepatocellular carcinoma. Nevertheless, the focus of effective management strategies is on lifestyle modification, for example dietary changes and physical activity, and novel pharmacological treatments of specific metabolic pathways (3).

The pathogenesis and therapeutic management of MAFLD depends on dietary regimens. Significantly increased hepatic lipid accumulation and progression of MAFLD are associated with dietary saturated fatty acids, sugars and refined carbohydrates. On the other hand, a diet high in omega-3 fatty acids, dietary fiber and antioxidants can attenuate hepatic lipid accumulation and inflammation (4). The Mediterranean diet, consisting of a lot of fruits, vegetables, whole grains and unsaturated fatty acids is particularly good for hepatic health (5). Akbulut et al. (6) in a systematic review found that this dietary pattern is useful in improving hepatic enzyme levels and hepatic steatosis (6). Both caloric restriction (or weight reduction) consistently reduce hepatic steatosis and improve metabolic parameters, and therefore both are recommended for managing MAFLD. Consequently, interventions aimed at preventing and treating MAFLD must be aimed at the diet (7).

A novel nutritional assessment instrument called the LLDS is developed to assess dietary adherence to a diet that promotes longevity and decreases the risk of chronic disease (8). The LLDS is grounded in extensive research that uses scientific evidence to quantify dietary intake patterns associated with specific nutrients and foods and health outcomes. This scoring system is focused on plant based foods, whole grains, nuts, lean protein sources, and away from processed foods, sugars, and red meat (9). Especially in clinical settings the LLDS is helpful as it enables the provision of individualized dietary counseling to nutritionists and physicians as well as monitoring of diet change over time (10). Studies have shown that higher scores of the LLDS are related to lower risk of metabolic disorders, cardiovascular disease, and cancer. The LLDS allows the individual to make informed dietary choices that promote longevity, and that prioritize preventive healthcare (11).

The rationale behind the study of the relationship between the LLDS and MAFLD is the rising prevalence of MAFLD and its potential for severe health consequences. MAFLD pathogenesis is well established to be associated with dietary habits (12). The LLDS (which gauges adherence to a dietary regimen to promote longevity and decrease the risk for chronic disease) is structured to study how adherence to a diet promoting longevity and decreasing the risk for chronic disease relates to hepatic health. Meta analysis has shown that dietary patterns have a powerful effect on metabolic health and hepatic lipid accumulation (13). Therefore, knowledge of the predictive value of LLDS adherence to MAFLD prevalence and severity could improve our knowledge of which dietary interventions attenuate hepatic injury and improve patient outcomes (14). This study may fill a critical gap in current metabolic health management strategies by validating the LLDS as a preventive strategy against MAFLD (15).

While genetic and proteomic factors also contribute to MAFLD susceptibility, diet remains a modifiable risk factor with direct implications for prevention. Given the multifactorial nature of MAFLD, evaluating diet in conjunction with known confounders provides a more accurate understanding of risk patterns. To date, few studies have specifically examined the LLDS—a unique diet quality index—in relation to MAFLD, warranting investigation into its potential role in disease modulation.

Given the emergence of the LLDS as a novel evidence-based scoring system for evaluating diet quality and the insufficient exploration of its relationship with MAFLD, this study aims to investigate the association between LLDS and MAFLD.

## 2 Methods

### 2.1 Study population

This case–control study was conducted at the King Khalid University Hospital, focusing on patients attending the Liver and Gastroenterology Clinic. The research enrolled 215 individuals recently diagnosed with MAFLD and 430 healthy controls. All participants were aged between 20 and 60 years, with data collection occurring from February 2023 to January 2025. In this study, MAFLD was diagnosed based on the presence of hepatic steatosis along with metabolic dysfunction. Hepatic steatosis was confirmed by abdominal ultrasonography performed by trained radiologists, demonstrating characteristic findings of fatty liver (hepatorenal echo contrast, vessel blurring, or deep attenuation), or elevated liver enzymes (ALT >30 U/L in men or >19 U/L in women; AST > 30 U/L in men or >25 U/L in women) in the absence of other liver diseases. Participants were required to meet at least one of the following metabolic criteria: overweight/obesity defined as BMI ≥ 23 kg/m^2^ (Asian-specific cutoff), type 2 diabetes mellitus (fasting glucose ≥126 mg/dL, HbA1c ≥ 6.5%, or use of antidiabetic medications), or evidence of at least two metabolic risk abnormalities including waist circumference ≥90 cm (men) or ≥80 cm (women), blood pressure ≥130/85 mmHg or antihypertensive treatment, fasting triglycerides ≥150 mg/dL or lipid-lowering therapy, HDL cholesterol <40 mg/dL (men) or <50 mg/dL (women), or prediabetes (fasting glucose 100–125 mg/dL or HbA1c 5.7–6.4%). Significant alcohol consumption (≥30 g/day for men or ≥20 g/day for women) and other chronic liver diseases such as viral hepatitis (negative HBsAg/anti-HCV), autoimmune hepatitis, or use of steatogenic medications were excluded. The control group consisted of healthy subjects who showed no indications of fatty liver disease through clinical assessment and liver ultrasound imaging. Participants with particular dietary patterns due to health issues or weight reduction were excluded from the study, along with those suffering from specific medical conditions such as kidney and liver diseases (including viral infections, autoimmune liver disorders, hemochromatosis, Wilson’s disease, and alcoholic fatty liver disease), cardiovascular issues, diabetes, cancers, thyroid disorders, and autoimmune diseases. Furthermore, individuals using medications that could adversely affect liver function or lead to weight gain were also excluded. The research additionally excluded individuals who answered fewer than 35 items on the food frequency questionnaire or inaccurately reported their daily caloric intake (below 800 kcal or above 4,500 kcal daily), but these individuals were substituted with others. Prior to their inclusion in the study, all participants gave informed written consent.

### 2.2 Dietary intake assessment

The dietary intake of the participants was assessed through the utilization of a validated semi-quantitative food frequency questionnaire, which comprised a total of 168 distinct food items. Participants were instructed to report their average consumption of various food items over the course of the previous year by selecting one of several predefined options. These options included: never or less than once a month, three to four times a month, once a week, two to four times a week, five to six times a week, once a day, two to three times a day, four to five times a day, or six times or more each day. Finally, the calculated daily intake was input into Nutritionist IV software to determine total energy and nutrient intake (16).

### 2.3 Lifelines diet score

The LLDS, an instrument for assessing individuals based on the quality of their diet, was derived using the methodology established by Vinke et al. (17). In line with the LLDS criteria, food categories were designated as having beneficial, neutral, detrimental, or uncertain health implications. The nine categories recognized for their positive health contributions encompass legumes and nuts, whole grain items, fruits, vegetables, fish, unsweetened dairy products, coffee and tea, soft margarine, and various oils. Conversely, butter and hard margarine, sugar-sweetened drinks, along with red and processed meats, represent three categories that adversely affect health. The food intake of each participant was quantified in grams per 1,000 kilocalories (kcal) and subsequently categorized into quintiles ranging from 1 to 5 points. Individuals in the top quintile for each beneficial food category received 5 points, while those in the lowest quintile were assigned 1 point. Conversely, for food categories that adversely affect health, a score of 1 indicates the highest intake, and a score of 5 denotes the lowest. The total scores for the consumption of 12 food categories comprising LLDS varied from 12 to 60 points (17). For example, a participant consuming high amounts of vegetables, whole grains, and fish but minimal processed meats and sugary beverages might score around 50–55 out of 60. Conversely, a diet rich in red meats and sugary drinks but low in plant-based foods might score around 20–25.

### 2.4 Covariate measurements

All anthropometric measurements were conducted in strict accordance with established standard protocols and were performed by a trained and qualified investigator. In the current study, height and weight measurements were performed by trained staff using standardized protocols. Participants’ weight was measured to the nearest 0.1 kg using a calibrated digital scale (SECA, Germany) while wearing light clothing and no shoes. Height was measured to the nearest 0.1 cm using a wall-mounted stadiometer (SECA, Germany) with participants standing barefoot, feet together, and head in the Frankfort horizontal plane position. All measurements were taken twice, and the average values were used for analysis. Body mass index (BMI) was calculated as weight (kg) divided by height squared (m^2^). These measurements were conducted under controlled conditions while adhering to all research standards. Additionally, waist circumference was measured in centimeters using a tape measure that has an accuracy of 0.5 centimeters. This measurement was taken at the midpoint located between the lower rib and the iliac crest, specifically at the end of a normal exhalation while the individual was standing upright. Furthermore, data regarding physical activity levels were assessed utilizing the International Physical Activity Questionnaire-Short Form (IPAQ-SH) (18), and the results were expressed in terms of metabolic equivalents calculated in minutes per week (MET-min/wk).

### 2.5 Assessment of other covariates

The necessary information, which encompasses various aspects such as demographic data, age, the presence of family History of disease, marital status, was obtained through the utilization of validated self-administered questionnaires specifically designed for this purpose.

### 2.6 Statistical analysis

Characteristics of participants were analyzed quantitatively and qualitatively using an independent t-test and chi-square test, respectively. The ANOVA test assessed dietary intakes across tertiles of LLDS. Logistic regression was employed to investigate the relationship between the Lifelines diet and MAFLD in both adjusted and unadjusted models. Model I accounted for energy intake, while further adjustments included marital status, waist circumference (WC), medical history, and physical activity. Model III also included body mass index (BMI) as an additional adjustment. A *p*-value of less than 0.05 was deemed statistically significant. Data analysis was conducted using the Statistical Package for Social Sciences (SPSS) version 26.0 (SPSS Inc., Chicago, Illinois, USA).

## 3 Results

The general characteristics and physical activity levels of participants with and without MAFLD are summarized in Table 1. The data indicate that there were no statistically significant differences in age, Body Mass Index (BMI), physical activity, waist circumference (WC), marital status, or history of disease between the case and control groups (*p* > 0.05). Additionally, the mean LLDS in the control group (39.94 ± 6.67) was significantly higher than that in the case group (32.84 ± 5.82) (*p* < 0.001). Table 2 presents the attributes of the study population segmented by tertiles of the LLDS. Following the tertiles of LLDS scores, no notable differences were found in age, gender, BMI, WC, physical activity, marital status, and Smoking among the tertiles of the LLDS scores (*p* > 0.05).

**Table 1:** The general characteristics of participants with and without MAFLD.

Variables	MAFLD (*n* = 215)	controls (*n* = 430)	*p*-value
Age (years)	36.23 ± 7.13	38.65 ± 7.92	0.233
Gender (female) *n* (%)	113 (52.5)	218 (50.7)	0.172
BMI (Kg/m^2^)	28.32 ± 4.61	27.03 ± 4.12	0.128
WC (cm)	103.74 ± 10.77	96.74 ± 9.62	0.087
Marital status (married) *n* (%)	181 (84.1)	382 (88.8)	0.314
Physical activity (MET-min/wk.)	1423.74 ± 751.70	1492.43 ± 792.64	0.091
Smoking, (yes) *n* (%)	41 (19.1)	75 (17.4)	0.134
Family History of disease, (yes) *n* (%)	69 (32.1)	127 (29.5)	0.413
LLDS (mean ± SD)	32.84 ± 5.82	39.94 ± 6.67	< 0.001

BMI, body mass index; MET, metabolic equivalent; WC, waist circumference, LLDS, Lifeline Diet Score. For quantitative variables mean ± SD; and for qualitative variables frequency (%) were used. *Independent t-test for quantitative variables and x^2^ test for categorical variables conducted.

**Table 2:** Characteristics of the study participants across tertiles of LLDS.

Variables	Tertiles of the LLDS score			*p*-value
	T1 (*n* = 226)	T2 (*n* = 218)	T3 (*n* = 201)	
Age (years)	36.01 ± 7.38	38.01 ± 7.16	38.21 ± 7.09	0.474
Gender (female) *n* (%)	116 (51.3)	110 (50.4)	106 (52.7)	0.352
BMI (Kg/m^2^)	28.63 ± 4.85	27.51 ± 4.57	27.47 ± 4.21	0.621
WC (cm)	103.18 ± 12.99	99.40 ± 11.33	96.20 ± 10.73	0.079
Marital status (married) *n* (%)	195 (86.3)	189 (87.1)	179 (89.4)	0.618
Physical activity (MET-min/wk.)	1522.69 + 838.71	1417.80 + 716.41	1445.19 + 768.24	0.523
Smoking, (yes) *n* (%)	44 (19.54)	40 (18.37)	35 (17.6)	0.103

Table 3 presents the energy and dietary consumption of study participants categorized by tertiles of LLDS scores. Those in the highest LLDS tertile exhibited markedly higher intakes of carbohydrates, calcium, magnesium, and folate (*p* < 0.001), while showing significantly lower levels of energy, fat, saturated fatty acids, and monounsaturated fatty acids (p < 0.001). There were no significant differences in the intake of protein, polyunsaturated fatty acids, vitamin B6, and vitamin B12 across the tertiles of LLDS. According to food group consumption, individuals scoring higher on the LLDS exhibited a notably increased consumption of vegetables, whole grains, legumes, nuts, fish, oils, and soft margarine, as well as coffee and unsweetened dairy. Conversely, their intake of red and processed meats, butter, hard margarine, and sugar-sweetened drinks was significantly lower (*p* < 0.001).

**Table 3:** Dietary intakes of study participants based on tertiles of LLDS.

Variables*	Tertiles of the LLDS score			*p*-value^**^
	T1 (*n* = 226)	T2 (*n* = 218)	T3 (*n* = 201)	
Energy intake (kcal)	2298.14 ± 685.79	2123.32 ± 603.12	1864.25 ± 547.45	<0.001
Carbohydrate (% of total daily energy)	56.52 ± 0.07	59.87 ± 0.06	63.22 ± 0.05	<0.001
Protein (% of total daily energy)	15.89 ± 0.03	15.65 ± 0.02	16.22 ± 0.02	0.062
Fat (% of total daily energy)	32.86 ± 0.05	28.64 ± 0.06	25.31 ± 0.05	<0.001
Cholesterol (mg/100Kcal)	117.56 ± 48.62	113.25 ± 50.75	116.10 ± 51.01	0.203
SFA (gr/1000Kcal)	11.21 ± 2.42	9.87 ± 2.46	8.13 ± 2.32	<0.001
MUFA (gr/1000Kcal)	11.11 ± 2.54	9.32 ± 2.24	7.61 ± 1.53	<0.001
PUFA (gr/1000Kcal)	9.82 ± 5.78	11.34 ± 7.90	12.65 ± 7.47	0.061
Calcium (mg/1000Kcal)	321.80 ± 98.74	402.08 ± 119.99	423.93 ± 103.85	<0.001
Magnesium (mg/1000Kcal)	117.67 ± 16.54	130.41 ± 20.38	139.85 ± 21.18	<0.001
Vitamin B6 (mg/1000Kcal)	0.79 ± 0.25	0.83 ± 0.24	0.88 ± 0.29	0.187
Folate (μg/1000Kcal)	124.48 ± 25.51	141.77 ± 35.11	159.53 ± 36.70	<0.001
B12 (μg/1000Kcal)	1.95 ± 0.84	1.78 ± 0.67	1.87 ± 0.58	0.414
Food groups				
Vegetables (gr/1000Kcal)	75.42 ± 48.97	109.11 ± 61.49	169.18 ± 79.17	<0.001
Fruits (gr/1000Kcal)	325.03 ± 134.68	333.12 ± 141.67	352.20 ± 151.69	0.076
Whole grain products (gr)	8.57 ± 10.44	26.85 ± 34.34	51.32 ± 45.13	<0.001
Legumes and nuts (gr/1000Kcal)	17.61 ± 7.90	22.85 ± 12.01	29.03 ± 13.41	<0.001
Fish (gr)	3.51 ± 3.16	4.15 ± 3.43	6.82 ± 4.65	<0.001
Oils and soft margarines (gr)	2.99 ± 2.73	4.41 ± 3.29	5.92 + 2.86	<0.001
Unsweetened dairy (gr)	88.30 ± 74.49	127.74 ± 91.09	138.22 ± 77.47	0.001
Coffee (gr)	4.10 ± 5.15	5.87 ± 15.742	8.12 ± 16.87	<0.001
Tea (gr)	171.20 ± 159.32	201.56 ± 306.23	196.79 ± 284.63	0.274
Red and processed meat (gr)	35.90 ± 18.26	23.97 ± 15.16	16.99 ± 14.25	<0.001
Butter and hard margarines	5.44 ± 5.79	3.54 ± 4.39	1.03 ± 1.31	<0.001
Sugar sweetened beverages(gr)	55.59 ± 56.12	23.55 ± 32.08	17.25 ± 30.51	0.001

MUFA, Monounsaturated Fatty Acid; PUFA, Ponounsaturated Fatty Acid; SFA, Saturated Fatty Acid; LLDS, lifelines diet score. Data reported on mean± standard deviation (SD). Statistically significant difference (*p*-value < 0.05).

** Obtained from one way ANOVA.

Odds ratio and 95% confidence interval for occurrence of the MAFLD across tertiles of LLDS are presented in Table 4. Participants with MAFLD in the highest LLDS tertile exhibited a 78% reduction in the odds of MAFLD compared to those in the lowest tertile (OR: 0.22; 95% CI: 0.12 to 0.36, *p* for trend: <0.001). This relationship remained statistically significant even after controlling for energy intake, age, gender, BMI, WC, family history, marital status, physical activity and smoking (OR: 0.19; 95% CI: 0.10 to 0.32, *p* for trend: <0.001).

**Table 4:** Odds ratio and 95% confidence interval for occurrence of the MAFLD across tertiles of LLDS.

MAFLD	Tertiles of the LLDS score			
	T1	T2	T3	*p* for trend
Crude model	1.00 (Ref)	0.38 (0.20–73)	0.22 (0.12–0.36)	<0.0001
Model 1^*^	1.00 (Ref)	0.44 (0.23–0.82)	(0.10–0.33) 0.20	<0.0001
Model 2^†^	1.00 (Ref)	0.41 (0.22–0.81)	0.21 (0.11–0.34)	<0.0001
Model 3^#^	1.00 (Ref)	0.43 (0.22–0.86)	0.19 (0.10–0.32)	<0.0001

* Model 1: adjusted for energy intake.

† Model 2: additionally, adjusted for age and gender.

# Model 3: additionally, adjusted for BMI, WC, family history, marital status, physical activity and smoking.

## 4 Discussion

This case–control study investigated the protective effect of the LLDS against MAFLD. Participants ranged from 20 to 60 years, with a recent MAFLD diagnosis or as healthy controls. The study found a significant inverse relationship between higher LLDS and lower MAFLD risk, based on the comparison between LLDS tertiles among all 645 participants (215 MAFLD patients vs. 430 controls). The findings suggest a diet high in LLDS is protective against MAFLD, though causal relationships require further study.

Recently, there has been interest in the association between diet quality and metabolic health outcomes, particularly with the LLDS and MAFLD. Women with higher adherence to LLDS had lower odds of metabolically unhealthy obesity (19) and overweight and obese adults with higher adherence to LLDS had reduced risk of metabolic syndrome (20). A second case–control study examined the effect of different dietary quality indicators on MAFLD. Significantly lower odds of MAFLD were associated with higher DDS and AHEI scores (21). In the study of another case–control study, the association between the LLDS and PCOS was investigated; significantly lower odds of PCOS were associated with higher LLDS scores (22). Significant correlation of MAFLD severity with dietary factors, including fruit intake, platelet count and diabetes mellitus, was found (23). Heredia et al. (24) found higher adherence to the Alternate Mediterranean Diet Score was associated with lower risk of MAFLD, but this association was mediated by BMI and total energy intake. In veterans, the Alternate Mediterranean Diet Score was inversely associated with MAFLD, but this relationship was mediated by BMI and total energy intake (24). Additionally, healthy low carbohydrate, low fat diets were protective against MAFLD, while unhealthy low fat diets increased MAFLD risk (25). Dietary and lifestyle inflammation scores were inversely associated with risk of MAFLD (26), also suggesting that pro-inflammatory diets and lifestyles may increase risk of MAFLD.

Moreover, recent studies have compared various dietary indices and their associations with MAFLD. For instance, Asiaei et al. demonstrated that the LLDS was inversely associated with NAFLD and visceral adiposity, particularly in women, suggesting the utility of LLDS in assessing diet-related liver health (27). In contrast, indices such as the Dietary Inflammatory Index (DII) and Empirical Dietary Inflammatory Potential (EDIP), which assess the pro- or anti-inflammatory potential of the diet, have also been strongly associated with MAFLD risk, especially among men (28). These indices emphasize the role of systemic inflammation as a key mechanism linking diet and liver steatosis. Another study focused on older adults found a positive association between nutritional status indices like the Prognostic Nutritional Index (PNI) and Geriatric Nutritional Risk Index (GNRI) with NAFLD prevalence, whereas the CONUT index showed an inverse association (29). While LLDS captures a broader view of dietary quality, the DII/EDIP reflect inflammatory dietary potential, and GNRI/PNI provide clinical insight into nutritional and immune status. Together, these findings highlight the complementary roles of various indices and suggest that integrating LLDS with other nutrition-based or inflammation-based indices may improve dietary assessment for MAFLD prevention and management.

Unlike previous research focusing on broader dietary indices or isolated nutrients, this study is among the first to directly investigate LLDS and its association with MAFLD risk. By utilizing both crude and adjusted models, and focusing on LLDS tertile rather than single dietary components, the study contributes novel insights into the utility of LLDS as a predictive and preventive tool in liver-related metabolic disorders.

The LLDS is unique in its linkage to MAFLD in contrast to the Mediterranean Diet (MD) and the Western Diet (30). The LLDS is primarily a diet of whole grains, fruits, vegetables and lean proteins, which is predominantly the same as the Mediterranean Diet, which is well known for its heart and metabolic benefits (31). Both diets have been shown to reduce risk factor for MAFLD by improving lipid profiles and insulin sensitivity, according to research. But the LLDS tends to include a wider range of whole foods and a more rigid constraint on processed foods and red meats, which may provide a small advantage in preventing the buildup of fat in the liver (32). Conversely, the Western Diet, including high intakes of red meats, processed foods, and sugary beverages, has been consistently associated with higher prevalence of MAFLD. This diet is shown in studies to worsen insulin resistance and increase hepatic fat storage, raising the risk of MAFLD (33). The protective effect of the LLDS against MAFLD is more substantial than that of the LLDS against the other diseases, suggesting that dietary intervention can play a more important role in promoting liver health and preventing disease progression (34). This comparative analysis shows that the LLDS is a superior dietary strategy for MAFLD management and prevention, compared to other common dietary patterns.

The mechanisms by which the LLDS may regulate MAFLD are multifaceted, and involve effects on the metabolic regulation and inflammatory processes (35). First, the LLDS encourages a lot of dietary fiber and antioxidants, which are found in abundance in fruits, vegetables and whole grains. Therefore, these components are important to improve insulin sensitivity and decrease systemic insulin levels which directly mitigate one of the most important drivers of MAFLD; insulin resistance (36). The second is that the LLDS focuses on eating lean protein sources and healthy fats — such as omega 3 fatty acids from fish and monounsaturated fats from olive oil. Modulating lipid profiles by reducing serum triglycerides and low density lipoprotein (LDL) cholesterol and increasing high density lipoprotein (HDL) cholesterol are these nutrients. Prevention of hepatic fat accumulation, a hallmark of MAFLD, requires this lipid modulation (37). In addition, the LLDS reduces the intake of processed foods and added sugars, known to further exacerbate hepatic steatosis and inflammation. The LLDS limits these dietary components to reduce caloric overload and oxidative stress on liver cells and prevent the progression of simple steatosis to more severe forms of MAFLD, such as steatohepatitis and fibrosis (38). Taken together, the LLDS addresses key metabolic derangements and reduces inflammatory triggers that contribute to the pathogenesis of MAFLD, and may represent a therapeutic dietary approach to treat this condition (39).

Results from this study strongly suggest that reducing dietary quality, as measured by the LLDS, is strongly associated with a significantly reduced risk of MAFLD. This translates clinically that dietary intervention targeting increased intake of beneficial (vegetables, whole grains, legumes, nuts, fish, etc.) and decreased intake of harmful (red and processed meats, sugary drinks, etc.) foods are likely to be useful in the prevention and treatment of MAFLD. Assessment of dietary quality and provision of personalized dietary counseling is a potential use of the LLDS. To validate the effectiveness of LLDS based interventions in diverse populations, further research is needed. The LLDS-based dietary recommendations could be easily integrated into routine clinical practice to considerably affect MAFLD prevention and management strategies.

Limitations of the design and methodology of this case control study investigating the association between the LLDS and MAFLD are highlighted, alongside the case for several strengths of the study. Strengths are the large sample size facilitating more statistical power, use of the validated LLDS for precise dietary assessment, multivariate logistic regression adjusting for confounding factors of energy intake, body mass index, waist circumference, physical activity and medical history and stringent exclusion criteria decreasing the level of confounding factors. Nevertheless, limitations exist because of the inability of the case control design to definitely establish causality, the potential for recall bias in self-reported dietary data, the cross sectional design which precludes determination of temporal relationships, and limited generalizability of findings to populations other than the population studied in a specific hospital. While the results are convincing of association, these limitations necessitate additional research with longitudinal studies and diverse populations to confirm causality and to extend generalizability. While our study demonstrates a robust association between LLDS and MAFLD, we acknowledge that unmeasured genetic (e.g., PNPLA3 polymorphism) and proteomic factors may modify this relationship. Future studies incorporating genetic and molecular data could further clarify whether dietary interventions are equally effective across different genetic risk profiles.

## 5 Conclusion

Our study strengthens the evidence that adherence to a healthy dietary pattern (as measured by LLDS) is associated with a lower MAFLD risk, even after accounting for major confounders. However, further research integrating genetic and molecular data is needed to refine personalized dietary recommendations for MAFLD prevention.

## Data Availability

The raw data supporting the conclusions of this article will be made available by the authors, without undue reservation.
